# Nursing students’ stressors and coping strategies during their first clinical training: a qualitative study in the United Arab Emirates

**DOI:** 10.1186/s12912-024-01962-5

**Published:** 2024-05-11

**Authors:** Jacqueline Maria Dias, Muhammad Arsyad Subu, Nabeel Al-Yateem, Fatma Refaat Ahmed, Syed Azizur Rahman, Mini Sara Abraham, Sareh Mirza Forootan, Farzaneh Ahmad Sarkhosh, Fatemeh Javanbakh

**Affiliations:** 1 Department of Nursing, College of Health Sciences, University of Sharjah, POBox, Sharjah 272272 UAE; 2 Health Care Management, College of Health Sciences, University of Sharjah, Sharjah, United Arab Emirates

**Keywords:** Adaptation, Clinical practicums, Coping strategies, Nursing students, Stressors

## Abstract

**Background:**

Understanding the stressors and coping strategies of nursing students in their first clinical training is important for improving student performance, helping students develop a professional identity and problem-solving skills, and improving the clinical teaching aspects of the curriculum in nursing programmes. While previous research have examined nurses’ sources of stress and coping styles in the Arab region, there is limited understanding of these stressors and coping strategies of nursing students within the UAE context thereby, highlighting the novelty and significance of the study.

**Methods:**

A qualitative study was conducted using semi-structured interviews. Overall 30 students who were undergoing their first clinical placement in Year 2 at the University of Sharjah between May and June 2022 were recruited. All interviews were recorded and transcribed verbatim and analyzed for themes.

**Results:**

During their first clinical training, nursing students are exposed to stress from different sources, including the clinical environment, unfriendly clinical tutors, feelings of disconnection, multiple expectations of clinical staff and patients, and gaps between the curriculum of theory classes and labatories skills and students’ clinical experiences. We extracted three main themes that described students’ stress and use of coping strategies during clinical training: (1) managing expectations; (2) theory-practice gap; and (3) learning to cope. Learning to cope, included two subthemes: positive coping strategies and negative coping strategies.

**Conclusions:**

This qualitative study sheds light from the students viewpoint about the intricate interplay between managing expectations, theory practice gap and learning to cope. Therefore, it is imperative for nursing faculty, clinical agencies and curriculum planners to ensure maximum learning in the clinical by recognizing the significance of the stressors encountered and help students develop positive coping strategies to manage the clinical stressors encountered. Further research is required look at the perspective of clinical stressors from clinical tutors who supervise students during their first clinical practicum.

**Supplementary Information:**

The online version contains supplementary material available at 10.1186/s12912-024-01962-5.

## Background

Nursing education programmes aim to provide students with high-quality clinical learning experiences to ensure that nurses can provide safe, direct care to patients [1]. The nursing baccalaureate programme at the University of Sharjah is a four year program with 137 credits. The programmes has both theoretical and clinical components withs nine clinical courses spread over the four years The first clinical practicum which forms the basis of the study takes place in year 2 semester 2.

Clinical practice experience is an indispensable component of nursing education and links what students learn in the classroom and in skills laboratories to real-life clinical settings [2–4]. However, a gap exists between theory and practice as the curriculum in the classroom differs from nursing students’ experiences in the clinical nursing practicum [5]. Clinical nursing training places (or practicums, as they are commonly referred to), provide students with the necessary experiences to ensure that they become proficient in the delivery of patient care [6]. The clinical practicum takes place in an environment that combines numerous structural, psychological, emotional and organizational elements that influence student learning [7] and may affect the development of professional nursing competencies, such as compassion, communication and professional identity [8]. While clinical training is a major component of nursing education curricula, stress related to clinical training is common among students [9]. Furthermore, the nursing literature indicates that the first exposure to clinical learning is one of the most stressful experiences during undergraduate studies [8, 10]. Thus, the clinical component of nursing education is considered more stressful than the theoretical component. Students often view clinical learning, where most learning takes place, as an unsupportive environment [11]. In addition, they note strained relationships between themselves and clinical preceptors and perceive that the negative attitudes of clinical staff produce stress [12].

The effects of stress on nursing students often involve a sense of uncertainty, uneasiness, or anxiety. The literature is replete with evidence that nursing students experience a variety of stressors during their clinical practicum, beginning with the first clinical rotation. Nursing is a complex profession that requires continuous interaction with a variety of individuals in a high-stress environment. Stress during clinical learning can have multiple negative consequences, including low academic achievement, elevated levels of burnout, and diminished personal well-being [13, 14]. In addition, both theoretical and practical research has demonstrated that increased, continual exposure to stress leads to cognitive deficits, inability to concentrate, lack of memory or recall, misinterpretation of speech, and decreased learning capacity [15]. Furthermore, stress has been identified as a cause of attrition among nursing students [16].

Most sources of stress have been categorized as academic, clinical or personal. Each person copes with stress differently [17], and utilizes deliberate, planned, and psychological efforts to manage stressful demands [18]. Coping mechanisms are commonly termed adaptation strategies or coping skills. Labrague et al. [19] noted that students used critical coping strategies to handle stress and suggested that problem solving was the most common coping or adaptation mechanism used by nursing students. Nursing students’ coping strategies affect their physical and psychological well-being and the quality of nursing care they offer. Therefore, identifying the coping strategies that students use to manage stressors is important for early intervention [20].

Studies on nursing students’ coping strategies have been conducted in various countries. For example, Israeli nursing students were found to adopt a range of coping mechanisms, including talking to friends, engaging in sports, avoiding stress and sadness/misery, and consuming alcohol [21]. Other studies have examined stress levels among medical students in the Arab region. Chaabane et al. [15], conducted a systematic review of sudies in Arab countries, including Saudi Arabia, Egypt, Jordan, Iraq, Pakistan, Oman, Palestine and Bahrain, and reported that stress during clinical practicums was prevalent, although it could not be determined whether this was limited to the initial clinical course or occurred throughout clinical training. Stressors highlighted during the clinical period in the systematic review included assignments and workload during clinical practice, a feeling that the requirements of clinical practice exceeded students’ physical and emotional endurance and that their involvement in patient care was limited due to lack of experience. Furthermore, stress can have a direct effect on clinical performance, leading to mental disorders. Tung et al. [22], reported that the prevalence of depression among nursing students in Arab countries is 28%, which is almost six times greater than the rest of the world [22]. On the other hand, Saifan et al. [5], explored the theory-practice gap in the United Arab Emirates and found that clinical stressors could be decreased by preparing students better for clinical education with qualified clinical faculty and supportive preceptors.

The purpose of this study was to identify the stressors experienced by undergraduate nursing students in the United Arab Emirates during their first clinical training and the basic adaptation approaches or coping strategies they used. Recognizing or understanding different coping processes can inform the implementation of corrective measures when students experience clinical stress. The findings of this study may provide valuable information for nursing programmes, nurse educators, and clinical administrators to establish adaptive strategies to reduce stress among students going clinical practicums, particularly stressors from their first clinical training in different healthcare settings.

## Methods

### Design

A qualitative approach was adopted to understand clinical stressors and coping strategies from the perspective of nurses’ lived experience. Qualitative content analysis was employed to obtain rich and detailed information from our qualitative data. Qualitative approaches seek to understand the phenomenon under study from the perspectives of individuals with lived experience [23]. Qualitative content analysis is an interpretive technique that examines the similarities and differences between and within different areas of text while focusing on the subject [24]. It is used to examine communication patterns in a repeatable and systematic way [25] and yields rich and detailed information on the topic under investigation [23]. It is a method of systematically coding and categorizing information and comprises a process of comprehending, interpreting, and conceptualizing the key meanings from qualitative data [26].

### Setting and participants

This study was conducted after the clinical rotations ended in April 2022, between May and June in the nursing programme at the College of Health Sciences, University of Sharjah, in the United Arab Emirates. The study population comprised undergraduate nursing students who were undergoing their first clinical training and were recruited using purposive sampling. The inclusion criteria for this study were second-year nursing students in the first semester of clinical training who could speak English, were willing to participate in this research, and had no previous clinical work experience. The final sample consisted of 30 students.

### Research instrument

The research instrument was a semi structured interview guide. The interview questions were based on an in-depth review of related literature. An intensive search included key words in Google Scholar, PubMed like the terms “nursing clinical stressors”, “nursing students”, and “coping mechanisms”. Once the questions were created, they were validated by two other faculty members who had relevant experience in mental health. A pilot test was conducted with five students and based on their feedback the following research questions, which were addressed in the study.

1. How would you describe your clinical experiences during your first clinical rotations?
2. In what ways did you find the first clinical rotation to be stressful?
3. What factors hindered your clinical training?
4. How did you cope with the stressors you encountered in clinical training?
5. Which strategies helped you cope with the clinical stressors you encountered?

### Data collection

Semi-structured interviews were chosen as the method for data collection. Semi structured interviews are a well-established approach for gathering data in qualitative research and allow participants to discuss their views, experiences, attitudes, and beliefs in a positive environment [27]. This approach allows for flexibility in questioning thereby ensuring that key topics related to clinical learning stressors and coping strategies would be explored. Participants were given the opportunity to express their views, experiences, attitudes, and beliefs in a positive environment, encouraging open communication. These semi structured interviews were conducted by one member of the research team (MAS) who had a mental health background, and another member of the research team who attended the interviews as an observer (JMD). Neither of these researchers were involved in teaching the students during their clinical practicum, which helped to minimize bias. The interviews took place at the University of Sharjah, specifically in building M23, providing a familiar and comfortable environment for the participant. Before the interviews were all students who agreed to participate were provided with an explanation of the study’s purpose. The time and location of each interview were arranged. Before the interviews were conducted, all students who provided consent to participate received an explanation of the purpose of the study, and the time and place of each interview were arranged to accommodate the participants’ schedules and preferences. The interviews were conducted after the clinical rotation had ended in April, and after the final grades had been submitted to the coordinator. The timings of the interviews included the month of May and June which ensured that participants have completed their practicum experience and could reflect on the stressors more comprehensively. The interviews were audio-recorded with the participants’ consent, and each interview lasted 25–40 min. The data were collected until saturation was reached for 30 students. Memos and field notes were also recorded as part of the data collection process. These additional data allowed for triangulation to improve the credibility of the interpretations of the data [28]. Memos included the interviewers’ thoughts and interpretations about the interviews, the research process (including questions and gaps), and the analytic progress used for the research. Field notes were used to record the interviewers’ observations and reflections on the data. These additional data collection methods were important to guide the researchers in the interpretation of the data on the participants’ feelings, perspectives, experiences, attitudes, and beliefs. Finally, member checking was performed to ensure conformability.

### Data analysis

The study used the content analysis method proposed by Graneheim and Lundman [24]. According to Graneheim and Lundman [24], content analysis is an interpretive technique that examines the similarities and differences between distinct parts of a text. This method allows researchers to determine exact theoretical and operational definitions of words, phrases, and symbols by elucidating their constituent properties [29]. First, we read the interview transcripts several times to reach an overall understanding of the data. All verbatim transcripts were read several times and discussed among all authors. We merged and used line-by-line coding of words, sentences, and paragraphs relevant to each other in terms of both the content and context of stressors and coping mechanisms. Next, we used data reduction to assess the relationships among themes using tables and diagrams to indicate conceptual patterns. Content related to stress encountered by students was extracted from the transcripts. In a separate document, we integrated and categorized all words and sentences that were related to each other in terms of both content and context. We analyzed all codes and units of meaning and compared them for similarities and differences in the context of this study. Furthermore, the emerging findings were discussed with other members of the researcher team. The final abstractions of meaningful subthemes into themes were discussed and agreed upon by the entire research team. This process resulted in the extraction of three main themes in addition to two subthemes related to stress and coping strategies.

### Ethical considerations

The University of Sharjah Research Ethics Committee provided approval to conduct this study (Reference Number: REC 19-12-03-01-S). Before each interview, the goal and study procedures were explained to each participant, and written informed consent was obtained. The participants were informed that participation in the study was voluntary and that they could withdraw from the study at any time. In the event they wanted to withdraw from the study, all information related to the participant would be removed. No participant withdrew from the study. Furthermore, they were informed that their clinical practicum grade would not be affected by their participation in this study. We chose interview locations in Building M23that were private and quiet to ensure that the participants felt at ease and confident in verbalizing their opinions. No participant was paid directly for involvement in this study. In addition, participants were assured that their data would remain anonymous and confidential. Confidentiality means that the information provided by participants was kept private with restrictions on how and when data can be shared with others. The participants were informed that their information would not be duplicated or disseminated without their permission. Anonymity refers to the act of keeping people anonymous with respect to their participation in a research endeavor. No personal identifiers were used in this study, and each participant was assigned a random alpha-numeric code (e.g., P1 for participant 1). All digitally recorded interviews were downloaded to a secure computer protected by the principal investigator with a password. The researchers were the only people with access to the interview material (recordings and transcripts). All sensitive information and materials were kept secure in the principal researcher’s office at the University of Sharjah. The data will be maintained for five years after the study is completed, after which the material will be destroyed (the transcripts will be shredded, and the tapes will be demagnetized).

## Results

In total, 30 nursing students who were enrolled in the nursing programme at the Department of Nursing, College of Health Sciences, University of Sharjah, and who were undergoing their first clinical practicum participated in the study. Demographically, 80% (*n* = 24) were females and 20% (*n* = 6) were male participants. The majority (83%) of study participants ranged in age from 18 to 22 years. 20% (*n* = 6) were UAE nationals, 53% (*n* = 16) were from Gulf Cooperation Council countries, while 20% (*n* = 6) hailed from Africa and 7% (*n* = 2) were of South Asian descent. 67% of the respondents lived with their families while 33% lived in the hostel. (Table 1)

**Table 1:** Demographic data participant descriptive statistics

**Gender**		
Female	24	80%
Male	6	20%
**Marital Status**		
Single	30	100%
Married	0	-
**Age**		
18–22	25	83%
23–27	5	17%
27 +	0	-
**Ethnicity**		
UAE Nationals	6	20%
GCC countries	16	53%
Africa	6	20%
South East Asia	2	7%
**Year of Study**		
Year 1	0	-
Year 2	30	100%
Year 3	0	-
Year 4	0	-
**Residence**		
Living with family	20	67%
Living in hostel	10	33%

Following the content analysis, we identified three main themes: (1) managing expectations, (2) theory-practice gap and 3)learning to cope. Learning to cope had two subthemes: positive coping strategies and negative coping strategies. An account of each theme is presented along with supporting excerpts for the identified themes. The identified themes provide valuable insight into the stressors encountered by students during their first clinical practicum. These themes will lead to targeted interventions and supportive mechanisms that can be built into the clinical training curriculum to support students during clinical practice.

### Theme 1: managing expectations

In our examination of the stressors experienced by nursing students during their first clinical practicum and the coping strategies they employed, we identified the first theme as managing expectations.

The students encountered expectations from various parties, such as clinical staff, patients and patients’ relatives which they had to navigate. They attempted to fulfil their expectations as they progressed through training, which presented a source of stress. The students noted that the hospital staff and patients expected them to know how to perform a variety of tasks upon request, which made the students feel stressed and out of place if they did not know how to perform these tasks. Some participants noted that other nurses in the clinical unit did not allow them to participate in nursing procedures, which was considered an enormous impediment to clinical learning, as noted in the excerpt below:

“…Sometimes the nurses… They will not allow us to do some procedures or things during clinical. And sometimes the patients themselves don’t allow us to do procedures” (P5).

Some of the students noted that they felt they did not belong and felt like foreigners in the clinical unit. Excerpts from the students are presented in the following quotes;

“The clinical environment is so stressful. I don’t feel like I belong. There is too little time to build a rapport with hospital staff or the patient” (P22).

“… you ask the hospital staff for some guidance or the location of equipment, and they tell us to ask our clinical tutor …but she is not around … what should I do? It appears like we do not belong, and the sooner the shift is over, the better” (P18).

“The staff are unfriendly and expect too much from us students… I feel like I don’t belong, or I am wasting their (the hospital staff’s) time. I want to ask questions, but they have loads to do” (P26).

Other students were concerned about potential failure when working with patients during clinical training, which impacted their confidence. They were particularly afraid of failure when performing any clinical procedures.

“At the beginning, I was afraid to do procedures. I thought that maybe the patient would be hurt and that I would not be successful in doing it. I have low self-confidence in doing procedures” (P13).

The call bell rings, and I am told to answer Room No. XXX. The patient wants help to go to the toilet, but she has two IV lines. I don’t know how to transport the patient… should I take her on the wheelchair? My eyes glance around the room for a wheelchair. I am so confused …I tell the patient I will inform the sister at the nursing station. The relative in the room glares at me angrily … “you better hurry up”…Oh, I feel like I don’t belong, as I am not able to help the patient… how will I face the same patient again?” (P12).

Another major stressor mentioned in the narratives was related to communication and interactions with patients who spoke another language, so it was difficult to communicate.

“There was a challenge with my communication with the patients. Sometimes I have communication barriers because they (the patients) are of other nationalities. I had an experience with a patient [who was] Indian, and he couldn’t speak my language. I did not understand his language” (P9).

Thus, a variety of expectations from patients, relatives, hospital staff, and preceptors acted as sources of stress for students during their clinical training.

### Theme 2: theory-practice gap

Theory-practice gaps have been identified in previous studies. In our study, there was complete dissonance between theory and actual clinical practice. The clinical procedures or practices nursing students were expected to perform differed from the theory they had covered in their university classes and skills lab. This was described as a theory–practice gap and often resulted in stress and confusion.

“For example …the procedures in the hospital are different. They are different from what we learned or from theory on campus. Or… the preceptors have different techniques than what we learned on campus. So, I was stress[ed] and confused about it” (P11).

Furthermore, some students reported that they did not feel that they received adequate briefing before going to clinical training. A related source of stress was overload because of the volume of clinical coursework and assignments in addition to clinical expectations. Additionally, the students reported that a lack of time and time management were major sources of stress in their first clinical training and impacted their ability to complete the required paperwork and assignments:

“…There is not enough time…also, time management at the hospital…for example, we start at seven a.m., and the handover takes 1 hour to finish. They (the nurses at the hospital) are very slow…They start with bed making and morning care like at 9.45 a.m. Then, we must fill [out] our assessment tool and the NCP (nursing care plan) at 10 a.m. So, 15 only minutes before going to our break. We (the students) cannot manage this time. This condition makes me and my friends very stressed out. -I cannot do my paperwork or assignments; no time, right?” (P10).

“Stressful. There is a lot of work to do in clinical. My experiences are not really good with this course. We have a lot of things to do, so many assignments and clinical procedures to complete” (P16).

The participants noted that the amount of required coursework and number of assignments also presented a challenge during their first clinical training and especially affected their opportunity to learn.

“I need to read the file, know about my patient’s condition and pathophysiology and the rationale for the medications the patient is receiving…These are big stressors for my learning. I think about assignments often. Like, we are just focusing on so many assignments and papers. We need to submit assessments and care plans for clinical cases. We focus our time to complete and finish the papers rather than doing the real clinical procedures, so we lose [the] chance to learn” (P25).

Another participant commented in a similar vein that there was not enough time to perform tasks related to clinical requirements during clinical placement.

“…there is a challenge because we do not have enough time. Always no time for us to submit papers, to complete assessment tools, and some nurses, they don’t help us. I think we need more time to get more experiences and do more procedures, reduce the paperwork that we have to submit. These are challenges …” (P14).

There were expectations that the students should be able to carry out their nursing duties without becoming ill or adversely affected. In addition, many students reported that the clinical environment was completely different from the skills laboratory at the college. Exposure to the clinical setting added to the theory-practice gap, and in some instances, the students fell ill.

One student made the following comment:

“I was assisting a doctor with a dressing, and the sight and smell from the oozing wound was too much for me. I was nauseated. As soon as the dressing was done, I ran to the bathroom and threw up. I asked myself… how will I survive the next 3 years of nursing?” (P14).

### Theme 3: learning to cope

The study participants indicated that they used coping mechanisms (both positive and negative) to adapt to and manage the stressors in their first clinical practicum. Important strategies that were reportedly used to cope with stress were time management, good preparation for clinical practice, and positive thinking as well as engaging in physical activity and self-motivation.

“Time management. Yes, it is important. I was encouraging myself. I used time management and prepared myself before going to the clinical site. Also, eating good food like cereal…it helps me very much in the clinic” (P28).

“Oh yeah, for sure positive thinking. In the hospital, I always think positively. Then, after coming home, I get [to] rest and think about positive things that I can do. So, I will think something good [about] these things, and then I will be relieved of stress” (P21).

Other strategies commonly reported by the participants were managing their breathing (e.g., taking deep breaths, breathing slowly), taking breaks to relax, and talking with friends about the problems they encountered.

“I prefer to take deep breaths and breathe slowly and to have a cup of coffee and to talk to my friends about the case or the clinical preceptor and what made me sad so I will feel more relaxed” (P16).

“Maybe I will take my break so I feel relaxed and feel better. After clinical training, I go directly home and take a long shower, going over the day. I will not think about anything bad that happened that day. I just try to think about good things so that I forget the stress” (P27).

“Yes, my first clinical training was not easy. It was difficult and made me stressed out…. I felt that it was a very difficult time for me. I thought about leaving nursing” (P7).

I was not able to offer my prayers. For me, this was distressing because as a Muslim, I pray regularly. Now, my prayer time is pushed to the end of the shift” (P11).

“When I feel stress, I talk to my friends about the case and what made me stressed. Then I will feel more relaxed” (P26).

Self-support or self-motivation through positive self-talk was also used by the students to cope with stress.

“Yes, it is difficult in the first clinical training. When I am stress[ed], I go to the bathroom and stand in the front of the mirror; I talk to myself, and I say, “You can do it,” “you are a great student.” I motivate myself: “You can do it”… Then, I just take breaths slowly several times. This is better than shouting or crying because it makes me tired” (P11).

Other participants used physical activity to manage their stress.

“How do I cope with my stress? Actually, when I get stressed, I will go for a walk on campus” (P4).

“At home, I will go to my room and close the door and start doing my exercises. After that, I feel the negative energy goes out, then I start to calm down… and begin my clinical assignments” (P21).

Both positive and negative coping strategies were utilized by the students. Some participants described using negative coping strategies when they encountered stress during their clinical practice. These negative coping strategies included becoming irritable and angry, eating too much food, drinking too much coffee, and smoking cigarettes.

“…Negative adaptation? Maybe coping. If I am stressed, I get so angry easily. I am irritable all day also…It is negative energy, right? Then, at home, I am also angry. After that, it is good to be alone to think about my problems” (P12).

“Yeah, if I…feel stress or depressed, I will eat a lot of food. Yeah, ineffective, like I will be eating a lot, drinking coffee. Like I said, effective, like I will prepare myself and do breathing, ineffective, I will eat a lot of snacks in between my free time. This is the bad side” (P16).

“…During the first clinical practice? Yes, it was a difficult experience for us…not only me. When stressed, during a break at the hospital, I will drink two or three cups of coffee… Also, I smoke cigarettes… A lot. I can drink six cups [of coffee] a day when I am stressed. After drinking coffee, I feel more relaxed, I finish everything (food) in the refrigerator or whatever I have in the pantry, like chocolates, chips, etc” (P23).

These supporting excerpts for each theme and the analysis offers valuable insights into the specific stressors faced by nursing students during their first clinical practicum. These insights will form the basis for the development of targeted interventions and supportive mechanisms within the clinical training curriculum to better support students’ adjustment and well-being during clinical practice.

## Discussion

Our study identified the stressors students encounter in their first clinical practicum and the coping strategies, both positive and negative, that they employed. Although this study emphasizes the importance of clinical training to prepare nursing students to practice as nurses, it also demonstrates the correlation between stressors and coping strategies.The content analysis of the first theme, managing expectations, paves the way for clinical agencies to realize that the students of today will be the nurses of tomorrow. It is important to provide a welcoming environment where students can develop their identities and learn effectively. Additionally, clinical staff should foster an environment of individualized learning while also assisting students in gaining confidence and competence in their repertoire of nursing skills, including critical thinking, problem solving and communication skills [8, 15, 19, 30]. Another challenge encountered by the students in our study was that they were prevented from participating in clinical procedures by some nurses or patients. This finding is consistent with previous studies reporting that key challenges for students in clinical learning include a lack of clinical support and poor attitudes among clinical staff and instructors [31]. Clinical staff with positive attitudes have a positive impact on students’ learning in clinical settings [32]. The presence, supervision, and guidance of clinical instructors and the assistance of clinical staff are essential motivating components in the clinical learning process and offer positive reinforcement [30, 33, 34]. Conversely, an unsupportive learning environment combined with unwelcoming clinical staff and a lack of sense of belonging negatively impact students’ clinical learning [35].

The sources of stress identified in this study were consistent with common sources of stress in clinical training reported in previous studies, including the attitudes of some staff, students’ status in their clinical placement and educational factors. Nursing students’ inexperience in the clinical setting and lack of social and emotional experience also resulted in stress and psychological difficulties [36]. Bhurtun et al. [33] noted that nursing staff are a major source of stress for students because the students feel like they are constantly being watched and evaluated.

We also found that students were concerned about potential failure when working with patients during their clinical training. Their fear of failure when performing clinical procedures may be attributable to low self-confidence. Previous studies have noted that students were concerned about injuring patients, being blamed or chastised, and failing examinations [37, 38]. This was described as feeling “powerless” in a previous study [7, 12]. In addition, patients’ attitudes towards “rejecting” nursing students or patients’ refusal of their help were sources of stress among the students in our study and affected their self-confidence. Self-confidence and a sense of belonging are important for nurses’ personal and professional identity, and low self-confidence is a problem for nursing students in clinical learning [8, 39, 40]. Our findings are consistent with a previous study that reported that a lack of self-confidence was a primary source of worry and anxiety for nursing students and affected their communication and intention to leave nursing [41].

In the second theme, our study suggests that students encounter a theory-practice gap in clinical settings, which creates confusion and presents an additional stressors. Theoretical and clinical training are complementary elements of nursing education [40], and this combination enables students to gain the knowledge, skills, and attitudes necessary to provide nursing care. This is consistent with the findings of a previous study that reported that inconsistencies between theoretical knowledge and practical experience presented a primary obstacle to the learning process in the clinical context [42], causing students to lose confidence and become anxious [43]. Additionally, the second theme, the theory-practice gap, authenticates Safian et al.’s [5] study of the theory-practice gap that exists United Arab Emirates among nursing students as well as the need for more supportive clinical faculty and the extension of clinical hours. The need for better time availability and time management to complete clinical tasks were also reported by the students in the study. Students indicated that they had insufficient time to complete clinical activities because of the volume of coursework and assignments. Our findings support those of Chaabane et al. [15]. A study conducted in Saudi Arabia [44] found that assignments and workload were among the greatest sources of stress for students in clinical settings. Effective time management skills have been linked to academic achievement, stress reduction, increased creativity [45], and student satisfaction [46]. Our findings are also consistent with previous studies that reported that a common source of stress among first-year students was the increased classroom workload [19, 47]. As clinical assignments and workloads are major stressors for nursing students, it is important to promote activities to help them manage these assignments [48].

Another major challenge reported by the participants was related to communicating and interacting with other nurses and patients. The UAE nursing workforce and population are largely expatriate and diverse and have different cultural and linguistic backgrounds. Therefore, student nurses encounter difficulty in communication [49]. This cultural diversity that students encounter in communication with patients during clinical training needs to be addressed by curriculum planners through the offering of language courses and courses on cultural diversity [50].

Regarding the third and final theme, nursing students in clinical training are unable to avoid stressors and must learn to cope with or adapt to them. Previous research has reported a link between stressors and the coping mechanisms used by nursing students [51–53]. In particular, the inability to manage stress influences nurses’ performance, physical and mental health, attitude, and role satisfaction [54]. One such study suggested that nursing students commonly use problem-focused (dealing with the problem), emotion-focused (regulating emotion), and dysfunctional (e.g., venting emotions) stress coping mechanisms to alleviate stress during clinical training [15]. Labrague et al. [51] highlighted that nursing students use both active and passive coping techniques to manage stress. The pattern of clinical stress has been observed in several countries worldwide. The current study found that first-year students experienced stress during their first clinical training [35, 41, 55]. The stressors they encountered impacted their overall health and disrupted their clinical learning. Chaabane et al. [15] reported moderate and high stress levels among nursing students in Bahrain, Egypt, Iraq, Jordan, Oman, Pakistan, Palestine, Saudi Arabia, and Sudan. Another study from Bahrain reported that all nursing students experienced moderate to severe stress in their first clinical placement [56]. Similarly, nursing students in Spain experienced a moderate level of stress, and this stress was significantly correlated with anxiety [30]. Therefore, it is imperative that pastoral systems at the university address students’ stress and mental health so that it does not affect their clinical performance. Faculty need to utilize evidence-based interventions to support students so that anxiety-producing situations and attrition are minimized.

In our study, students reported a variety of positive and negative coping mechanisms and strategies they used when they experienced stress during their clinical practice. Positive coping strategies included time management, positive thinking, self-support/motivation, breathing, taking breaks, talking with friends, and physical activity. These findings are consistent with those of a previous study in which healthy coping mechanisms used by students included effective time management, social support, positive reappraisal, and participation in leisure activities [57]. Our study found that relaxing and talking with friends were stress management strategies commonly used by students. Communication with friends to cope with stress may be considered social support. A previous study also reported that people seek social support to cope with stress [58]. Some students in our study used physical activity to cope with stress, consistent with the findings of previous research. Stretching exercises can be used to counteract the poor posture and positioning associated with stress and to assist in reducing physical tension. Promoting such exercise among nursing students may assist them in coping with stress in their clinical training [59].

Our study also showed that when students felt stressed, some adopted negative coping strategies, such as showing anger/irritability, engaging in unhealthy eating habits (e.g., consumption of too much food or coffee), or smoking cigarettes. Previous studies have reported that high levels of perceived stress affect eating habits [60] and are linked to poor diet quality, increased snacking, and low fruit intake [61]. Stress in clinical settings has also been linked to sleep problems, substance misuse, and high-risk behaviors’ and plays a major role in student’s decision to continue in their programme.

### Implications of the study

The implications of the study results can be grouped at multiple levels including; clinical, educational, and organizational level. A comprehensive approach to addressing the stressors encountered by nursing students during their clinical practicum can be overcome by offering some practical strategies to address the stressors faced by nursing students during their clinical practicum. By integrating study findings into curriculum planning, mentorship programs, and organizational support structures, a supportive and nurturing environment that enhances students’ learning, resilience, and overall success can be envisioned.

### Clinical level

Introducing simulation in the skills lab with standardized patients and the use of moulage to demonstrate wounds, ostomies, and purulent dressings enhances students’ practical skills and prepares them for real-world clinical scenarios. Organizing orientation days at clinical facilities helps familiarize students with the clinical environment, identify potential stressors, and introduce interventions to enhance professionalism, social skills, and coping abilities Furthermore, creating a WhatsApp group facilitates communication and collaboration among hospital staff, clinical tutors, nursing faculty, and students, enabling immediate support and problem-solving for clinical situations as they arise, Moreover, involving chief nursing officers of clinical facilities in the Nursing Advisory Group at the Department of Nursing promotes collaboration between academia and clinical practice, ensuring alignment between educational objectives and the needs of the clinical setting [62].

### Educational level

Sharing study findings at conferences (we presented the results of this study at Sigma Theta Tau International in July 2023 in Abu Dhabi, UAE) and journal clubs disseminates knowledge and best practices among educators and clinicians, promoting awareness and implementation of measures to improve students’ learning experiences. Additionally we hold mentorship training sessions annually in January and so we shared with the clinical mentors and preceptors the findings of this study so that they proactively they are equipped with strategies to support students’ coping with stressors during clinical placements.

### Organizational level

At the organizational we relooked at the available student support structures, including counseling, faculty advising, and career advice, throughout the nursing program emphasizing the importance of holistic support for students’ well-being and academic success as well as retention in the nursing program. Also, offering language courses as electives recognizes the value of communication skills in nursing practice and provides opportunities for personal and professional development.

## Conclusions

For first-year nursing students, clinical stressors are inevitable and must be given proper attention. Recognizing nursing students’ perspectives on the challenges and stressors experienced in clinical training is the first step in overcoming these challenges. In nursing schools, providing an optimal clinical environment as well as increasing supervision and evaluation of students’ practices should be emphasized. Our findings demonstrate that first-year nursing students are exposed to a variety of different stressors. Identifying the stressors, pressures, and obstacles that first-year students encounter in the clinical setting can assist nursing educators in resolving these issues and can contribute to students’ professional development and survival to allow them to remain in the profession. To overcome stressors, students frequently employ problem-solving approaches or coping mechanisms. The majority of nursing students report stress at different levels and use a variety of positive and negative coping techniques to manage stress.

The present results may not be generalizable to other nursing institutions because this study used a purposive sample along with a qualitative approach and was limited to one university in the Middle East. Furthermore, the students self-reported their stress and its causes, which may have introduced reporting bias. The students may also have over or underreported stress or coping mechanisms because of fear of repercussions or personal reasons, even though the confidentiality of their data was ensured. Further studies are needed to evaluate student stressors and coping now that measures have been introduced to support students. Time will tell if these strategies are being used effectively by both students and clinical personnel or if they need to be readdressed. Finally, we need to explore the perceptions of clinical faculty towards supervising students in their first clinical practicum so that clinical stressors can be handled effectively.

### Electronic supplementary material

Below is the link to the electronic supplementary material.

Supplementary Material 1

## Data Availability

The data sets are available with the corresponding author upon reasonable request.
