# Supplementary material for: Nursing students’ stressors and coping strategies during their first clinical training: a qualitative study in the United Arab Emirates
Source: BMC Nurs. 2024 May 11;23:322. doi: 10.1186/s12912-024-01962-5 (PMC11088033; doi:10.1186/s12912-024-01962-5)
Supplement: Supplementary file 1 — Supplementary Material 1 [file 12912_2024_1962_MOESM1_ESM.docx]

**Multiple Expectations**

**Unpredictable Clinical Learning Environment**

**Theoretical Knowledge Did Not Translate Into Skills**

**Learning To Cope**

**Managing Expectations**

**Theory Practice Gap**

**Challenges**

**Bridging Theory Into Practice**

**Self-doubt**

**Engaging In Self Care**

**Positive Mindset**

**Exercise**

**Denial**

**Substance Abuse**

**Avoidance**

**Social Support**

**Negative Coping Mechanisms**

**Positive Coping Mechanisms**

**Feeling Overwhelmed**
